# Supplementary material for: Relationships of residential distance to greenhouse floriculture and organophosphate, pyrethroid, and neonicotinoid urinary metabolite concentration in Ecuadorian Adolescents
Source: Int J Health Geogr. 2025 Apr 18;24:9. doi: 10.1186/s12942-025-00395-w (PMC12008992; doi:10.1186/s12942-025-00395-w)
Supplement: Supplementary file 1 — Additional file 1. [file 12942_2025_395_MOESM1_ESM.docx]

Table S1. Percent difference (β% [95%CI]) of metabolite concentration for every 50% increase in distance from the home to the nearest greenhouse using a 100m, 150m, 250m, 300m, 400m, and 750m strata.

|  | **100m Strata** | | **150m Strata** | | **250m Strata** | | **300m Strata** | | **400m Strata** | | **750m Strata** | |
| --- | --- | --- | --- | --- | --- | --- | --- | --- | --- | --- | --- | --- |
| **Metabolite** | **≤100 M** | **>100 M** | **≤150 M** | **>150 M** | **≤250 M** | **>250 M** | **≤300 M** | **>300 M** | **≤400 M** | **>400 M** | **≤-750 M** | **>-750 M** |
| Organophosphate Summary Score | -0.69 (-3.07, 1.75) | 1.13 (-0.42, 2.71) | -0.94 (-2.86, 1.03) | 0.00 (-1.72, 1.75) | 1.04 (-1.59, 3.73) | 0.03 (-1.51, 1.60) | 0.00 (-1.72, 1.75) | 1.04 (-1.59, 3.73) | 0.03 (-1.51, 1.60) | -0.50 (-3.66, 2.77) | 0.34 (-0.84, 1.53) | -1.04 (-6.37, 4.59) |
| PNP | -0.97 (-4.39, 2.57) | 2.70 (0.71, 4.72)* | -2.42 (-5.03, 0.26) | -2.17 (-4.31, 0.01) | 3.92 (0.38, 7.58)* | -1.34 (-3.29, 0.65) | -2.17 (-4.31, 0.01) | 3.92 (0.38, 7.58)* | -1.34 (-3.29, 0.65) | 3.87 (-0.41, 8.33) | -1.09 (-2.61, 0.46) | 7.50 (0.14, 15.40)* |
| TCPy | 1.60 (-2.89, 6.30) | 2.36 (-0.66, 5.47) | 1.22 (-2.30, 4.86) | 1.54 (-1.72, 4.91) | 3.35 (-1.82, 8.80) | 1.55 (-1.43, 4.62) | 1.54 (-1.72, 4.91) | 3.35 (-1.82, 8.80) | 1.55 (-1.43, 4.62) | 2.80 (-3.49, 9.49) | 1.97 (-0.34, 4.34) | 4.61 (-6.20, 16.66) |
| MDA | -1.43 (-6.59, 4.01) | 0.07 (-2.86, 3.09) | -2.10 (-6.25, 2.23) | -0.14 (-3.65, 3.48) | -2.00 (-6.77, 3.01) | -0.26 (-3.32, 2.89) | -0.14 (-3.65, 3.48) | -2.00 (-6.77, 3.01) | -0.26 (-3.32, 2.89) | -4.96 (-10.63, 1.07) | 0.30 (-2.01, 2.66) | -4.03 (-14.15, 7.28) |
| IMPy |  |  |  |  |  |  |  |  |  |  |  |  |
| Neonicotinoid Summary Score | -2.04 (-7.74, 4.02) | 3.33 (-0.39, 7.18) | -3.66 (-8.33, 1.25) | -2.18 (-6.20, 2.01) | 1.82 (-4.38, 8.42) | -0.84 (-4.39, 2.85) | -2.18 (-6.20, 2.01) | 1.82 (-4.38, 8.42) | -0.84 (-4.39, 2.85) | -0.02 (-7.76, 8.36) | -0.76 (-3.50, 2.06) | 0.37 (-13.03, 15.85) |
| OHIM | -1.05 (-7.02, 5.30) | 0.42 (-3.21, 4.18) | -2.89 (-7.90, 2.39) | -0.90 (-5.43, 3.84) | 0.49 (-4.89, 6.17) | -1.41 (-5.26, 2.60) | -0.90 (-5.43, 3.84) | 0.49 (-4.89, 6.17) | -1.41 (-5.26, 2.60) | -0.91 (-7.53, 6.19) | -1.09 (-3.98, 1.89) | 3.96 (-8.00, 17.47) |
| AND | 4.92 (-3.92, 14.56) | 3.92 (-0.73, 8.78) | -0.41 (-7.11, 6.78) | -2.86 (-7.73, 2.26) | 1.03 (-7.01, 9.75) | -1.61 (-6.11, 3.10) | -2.86 (-7.73, 2.26) | 1.03 (-7.01, 9.75) | -1.61 (-6.11, 3.10) | 1.49 (-8.45, 12.51) | 0.42 (-3.20, 4.17) | 19.16 (0.99, 40.61)* |
| Pyrethroid Summary Score | 1.55 (-2.14, 5.38) | -1.30 (-3.40, 0.85) | 1.35 (-1.84, 4.65) | 0.99 (-1.57, 3.62) | -1.88 (-5.27, 1.63) | 0.56 (-1.67, 2.84) | 0.99 (-1.57, 3.62) | -1.88 (-5.27, 1.63) | 0.56 (-1.67, 2.84) | -4.39 (-8.44, -0.16)* | 0.58 (-1.11, 2.31) | 0.13 (-6.77, 7.55) |
| 3-PBA | 4.65 (-1.59, 11.29) | -2.48 (-6.13, 1.31) | 4.16 (-1.20, 9.81) | 0.88 (-3.41, 5.36) | -5.82 (-11.73, 0.49) | 0.66 (-3.09, 4.57) | 0.88 (-3.41, 5.36) | -5.82 (-11.73, 0.49) | 0.66 (-3.09, 4.57) | -9.82 (-16.80, -2.25)* | 1.20 (-1.70, 4.20) | -5.96 (-18.72, 8.80) |
| *trans-*DCCA | -0.57 (-6.07, 5.24) | -1.64 (-4.61, 1.43) | -0.26 (-4.97, 4.69) | 0.67 (-3.15, 4.64) | -0.31 (-4.97, 4.57) | -0.05 (-3.32, 3.32) | 0.67 (-3.15, 4.64) | -0.31 (-4.97, 4.57) | -0.05 (-3.32, 3.32) | -2.76 (-8.37, 3.19) | -0.09 (-2.54, 2.41) | 3.52 (-6.19, 14.24) |
| Metabolites outcomes are creatinine adjusted Models adjusted for age, height-for-age z-score, BMI-for-age z-score, race, gender, monthly income, parental education, living with an agricultural or flower worker. Values below the LOD and were imputed using LOD/√2. *p<0.05  m=meters, PNP= para-Nitrophenol, TCPy= 3,5,6-Trichloro-2-pyridinol, MDA= malathion dicarboxylic acid, IMPy= 2-isopropyl-4-methyl-6-hydroxypyrimidine, OHIM= 5-Hydroxy imidacloprid, AND=Acetamiprid-N-desmethyl, 3-PBA=3-phenoxybenzoic acid, trans-DCCA= trans-3-(2,2-Dichlorovinyl)-2,2-dimethylcyclopropane carboxylic acid | | | | | | | | | | | | |
