# Supplementary material for: Relationships of residential distance to greenhouse floriculture and organophosphate, pyrethroid, and neonicotinoid urinary metabolite concentration in Ecuadorian Adolescents
Source: Int J Health Geogr. 2025 Apr 18;24:9. doi: 10.1186/s12942-025-00395-w (PMC12008992; doi:10.1186/s12942-025-00395-w)
Supplement: Supplementary file 2 — Additional file 2. [file 12942_2025_395_MOESM2_ESM.docx]

Table S2. Percent difference of metabolite concentration per 50% increase in residential distance to the nearest floricultural greenhouse (β%_per 50% greater distance_ (95% CI)) using Tobit regression.

| **Metabolite** | **β% _per 50% greater distance_ (95% CI)** | **Standard Error** | **P-value** |
| --- | --- | --- | --- |
| IMPy | -1.83 | 3.18 | 0.56 |
| MDA | -1.12 | 1.65 | 0.49 |
| OHIM | -1.91 | 2.67 | 0.46 |
| AND | 0.49 | 2.98 | 0.87 |
| 3-PBA | -0.96 | 1.21 | 0.43 |
| *trans-DCCA* | -0.58 | 0.93 | 0.53 |
| *p<0.05  Models adjusted for age, height-for-age z-score, BMI-for-age z-score, race, gender, monthly income, parental education, living with an agricultural or flower worker.  MDA= malathion dicarboxylic acid, IMPy= 2-isopropyl-4-methyl-6-hydroxypyrimidine, OHIM= 5-Hydroxy imidacloprid, AND=Acetamiprid-N-desmethyl, 3-PBA=3-phenoxybenzoic acid, trans-DCCA= trans-3-(2,2-Dichlorovinyl)-2,2-dimethylcyclopropane carboxylic acid  The left bound censoring value was determined by taking the LOD, dividing by creatinine, and ln-transforming the value. | | | |
|  |  |  |  |
|  |  |  |  |
|  |  |  |  |
|  |  |  |  |
|  |  |  |  |
