# Supplementary material for: Relationships of residential distance to greenhouse floriculture and organophosphate, pyrethroid, and neonicotinoid urinary metabolite concentration in Ecuadorian Adolescents
Source: Int J Health Geogr. 2025 Apr 18;24:9. doi: 10.1186/s12942-025-00395-w (PMC12008992; doi:10.1186/s12942-025-00395-w)
Supplement: Supplementary file 3 — Additional file 3. [file 12942_2025_395_MOESM3_ESM.docx]

Table S3. Percent change (β% [95%CI]) of metabolite concentration for every 50% increase in surface area concentration within a 100m, 200m, 300m, 500m, and 750m buffer size.

| **Metabolite** | **100m** | **200m** | **300m** | **500m** | **750m** |
| --- | --- | --- | --- | --- | --- |
| Organophosphate Summary Score | -0.10 (-0.50, 0.31) | -0.18 (-0.47, 0.12) | -0.13 (-0.39, 0.14) | -0.15 (-0.39, 0.10) | -0.15 (-0.42, 0.13) |
| PNP | 0.32 (-0.20, 0.86) | -0.05 (-0.43, 0.34) | -0.12 (-0.46, 0.22) | -0.17 (-0.49, 0.15) | -0.31 (-0.66, 0.04) |
| TCPy | -0.71 (-1.48, 0.07) | -0.49 (-1.06, 0.08) | -0.45 (-0.95, 0.06) | -0.43 (-0.90, 0.05) | -0.53 (-1.05, -0.002)* |
| MDA | 0.01 (-0.79, 0.81) | -0.16 (-0.74, 0.42) | -0.05 (-0.57, 0.47) | -0.05 (-0.53, 0.44) | 0.08 (-0.45, 0.62) |
| IMPy | 0.65 (-0.32, 1.64) | -0.20 (-0.91, 0.51) | -0.09 (-0.72, 0.54) | -0.03 (-0.62, 0.57) | -0.01 (-0.66, 0.65) |
| Neonicotinoid Summary Score | 0.57 (-0.20, 1.34) | -0.14 (-0.59, 0.31) | -0.03 (-0.53, 0.47) | -0.01 (-0.48, 0.45) | 0.17 (-0.35, 0.68) |
| OHIM | 0.62 (-0.39, 1.63) | -0.04 (-0.95, 0.88) | 0.35 (-0.30, 1.00) | 0.23 (-0.38, 0.84) | 0.33 (-0.33, 1.01) |
| AND | 0.89 (-0.37, 2.16) | -0.24 (-0.72, 0.23) | -0.25 (-1.06, 0.56) | -0.24 (-1.00, 0.53) | 0.05 (-0.79, 0.90) |
| Pyrethroid Summary Score | 0.03 (-0.54, 0.61) | 0.04 (-0.38, 0.45) | 0.14 (-0.23, 0.51) | 0.14 (-0.21, 0.49) | 0.32 (-0.07, 0.70) |
| 3-PBA | 0.20 (-0.81, 1.23) | 0.29 (-0.45, 1.03) | 0.08 (-0.58, 0.73) | 0.12 (-0.49, 0.74) | 0.53 (-0.15, 1.21) |
| *trans-*DCCA | -0.04 (-0.85, 0.79) | 0.02 (-0.58, 0.62) | 0.30 (-0.23, 0.83) | 0.25 (-0.25, 0.75) | 0.30 (-0.25, 0.86) |
| Metabolites outcomes are creatinine adjusted Models adjusted for age, height-for-age z-score, BMI-for-age z-score, race, gender, monthly income, parental education, living with an agricultural or flower worker. 62.3% of MDA, 75.8% of IMPY, 71.2% of OHIM,62.2% of AND, 11.1% of 3-PBA, and 80.8% trans-DCCA had values below the LOD and were imputed using LOD/√2. *p<0.05  m=meters, PNP= para-Nitrophenol, TCPy= 3,5,6-Trichloro-2-pyridinol, MDA= malathion dicarboxylic acid, IMPy= 2-isopropyl-4-methyl-6-hydroxypyrimidine, OHIM= 5-Hydroxy imidacloprid, AND=Acetamiprid-N-desmethyl, 3-PBA=3-phenoxybenzoic acid, *trans*-DCCA= trans-3-(2,2-Dichlorovinyl)-2,2-dimethylcyclopropane carboxylic acid | | | | | |
