# Supplementary material for: Relationships of residential distance to greenhouse floriculture and organophosphate, pyrethroid, and neonicotinoid urinary metabolite concentration in Ecuadorian Adolescents
Source: Int J Health Geogr. 2025 Apr 18;24:9. doi: 10.1186/s12942-025-00395-w (PMC12008992; doi:10.1186/s12942-025-00395-w)
Supplement: Supplementary file 4 — Additional file 4. [file 12942_2025_395_MOESM4_ESM.docx]

Table S4. Percent difference of metabolite concentration for every 50% increase in surface area within 150m from homes (β% _per 50% greater area_ [95%CI]) using Tobit regression.

| **Metabolite** | **β% _per 50% greater area_ (95%CI)** | **Standard Error** | **P-value** |
| --- | --- | --- | --- |
| IMPy | 1.20 | 1.11 | 0.28 |
| MDA | -0.44 | 0.58 | 0.45 |
| OHIM | 0.25 | 0.95 | 0.79 |
| AND | 1.04 | 1.06 | 0.33 |
| 3-PBA | 0.41 | 0.44 | 0.35 |
| *trans-DCCA* | 0.08 | 0.34 | 0.81 |
| *p<0.05  Models adjusted for age, height-for-age z-score, BMI-for-age z-score, race, gender, monthly income, parental education, living with an agricultural or flower worker.  PNP= para-Nitrophenol, TCPy= 3,5,6-Trichloro-2-pyridinol, MDA= malathion dicarboxylic acid, IMPy= 2-isopropyl-4-methyl-6-hydroxypyrimidine, OHIM= 5-Hydroxy imidacloprid, AND=Acetamiprid-N-desmethyl, 3-PBA=3-phenoxybenzoic acid, trans-DCCA= trans-3-(2,2-Dichlorovinyl)-2,2-dimethylcyclopropane carboxylic acid  The left bound censoring value was determined by taking the LOD, dividing by creatinine, and ln-transforming the value. | | | |
|  |  |  |  |
|  |  |  |  |
|  |  |  |  |
|  |  |  |  |
|  |  |  |  |
