# Supplementary material for: Relationships of residential distance to greenhouse floriculture and organophosphate, pyrethroid, and neonicotinoid urinary metabolite concentration in Ecuadorian Adolescents
Source: Int J Health Geogr. 2025 Apr 18;24:9. doi: 10.1186/s12942-025-00395-w (PMC12008992; doi:10.1186/s12942-025-00395-w)
Supplement: Supplementary file 5 — Additional file 5. [file 12942_2025_395_MOESM5_ESM.docx]

Table S5. Moran’s I for linear regression residuals which assessed the association between home distance to the nearest greenhouse and urinary pesticide metabolite concentration.

|  | OLS Residuals | | |
| --- | --- | --- | --- |
|  | Moran I | z-score | p-value |
| Organophosphate Summary Score | 0.14 | 3.63 | <0.001 |
| PNP | 0.12 | 3.13 | <0.001 |
| TCPY | 0.19 | 5.20 | <0.001 |
| MDA | 0.13 | 3.57 | <0.001 |
| IMPY | 0.18 | 4.79 | <0.001 |
| Neonicotinoid Summary Score | 0.09 | 2.32 | 0.02 |
| OHIM | 0.11 | 3.10 | <0.001 |
| AND | 0.11 | 2.80 | 0.01 |
| Pyrethroid Summary Score | 0.16 | 4.21 | <0.001 |
| 3-PBA | 0.18 | 4.71 | <0.001 |
| *trans*-DCCA | 0.12 | 3.15 | <0.001 |
| PNP= para-Nitrophenol, TCPy= 3,5,6-Trichloro-2-pyridinol, MDA= malathion dicarboxylic acid, IMPy= 2-isopropyl-4-methyl-6-hydroxypyrimidine, OHIM= 5-Hydroxy imidacloprid, AND=Acetamiprid-N-desmethyl, 3-PBA=3-phenoxybenzoic acid, trans-DCCA= trans-3-(2,2-Dichlorovinyl)-2,2-dimethylcyclopropane carboxylic acid | | | |
